# ARGONAUT-III and -V: susceptibility of carbapenem-resistant *Klebsiella pneumoniae* and multidrug-resistant *Pseudomonas aeruginosa* to the bicyclic boronate β-lactamase inhibitor taniborbactam combined with cefepime

**DOI:** 10.1128/aac.00751-24

**Published:** 2024-08-12

**Authors:** Michael R. Jacobs, Ayman M. Abdelhamed, Caryn E. Good, Andrew R. Mack, Christopher R. Bethel, Steven Marshall, Andrea M. Hujer, Kristine M. Hujer, Robin Patel, David van Duin, Vance G. Fowler, Daniel D. Rhoads, David A. Six, Greg Moeck, Tsuyoshi Uehara, Krisztina M. Papp-Wallace, Robert A. Bonomo

**Affiliations:** 1 Case Western Reserve University, Cleveland, Ohio, USA; 2 University Hospitals Cleveland Medical Center, Cleveland, Ohio, USA; 3 Department of Molecular Biology and Microbiology, Case Western Reserve University School of Medicine, Cleveland, Ohio, USA; 4 Louis Stokes Cleveland Department of Veterans Affairs Medical Center, Research Service, Cleveland, Ohio, USA; 5 Department of Medicine, Case Western Reserve University School of Medicine, Cleveland, Ohio, USA; 6 Division of Clinical Microbiology, Department of Laboratory Medicine and Pathology, Mayo Clinic, Rochester, Minnesota, USA; 7 Division of Infectious Diseases, University of North Carolina, Chapel Hill, North Carolina, USA; 8 Duke Clinical Research Institute, Duke University Medical Center, Durham, North Carolina, USA; 9 Department of Pathology, Cleveland Clinic Lerner College of Medicine, Cleveland, Ohio, USA; 10 Venatorx Pharmaceuticals Inc., Malvern, Pennsylvania, USA; 11 Departments of Biochemistry, Pharmacology, Proteomics and Bioinformatics, Case Western Reserve University School of Medicine, Cleveland, Ohio, USA; 12 CWRU-Cleveland VAMC Center for Antimicrobial Resistance and Epidemiology (Case VA CARES), Cleveland, Ohio, USA; Universita degli studi di roma La Sapienza, Rome, Italy

**Keywords:** taniborbactam, *Klebsiella pneumoniae*, *Pseudomonas aeruginosa*, bicyclic boronate, beta-lactamase inhibitor, cefepime taniborbactam

## Abstract

Taniborbactam, a bicyclic boronate β-lactamase inhibitor with activity against *Klebsiella pneumoniae* carbapenemase (KPC), Verona integron–encoded metallo-β-lactamase (VIM), New Delhi metallo-β-lactamase (NDM), extended-spectrum beta-lactamases (ESBLs), OXA-48, and AmpC β-lactamases, is under clinical development in combination with cefepime. Susceptibility of 200 previously characterized carbapenem-resistant *K. pneumoniae* and 197 multidrug-resistant (MDR) *Pseudomonas aeruginosa* to cefepime-taniborbactam and comparators was determined by broth microdilution. For *K. pneumoniae* (192 KPC; 7 OXA-48-related), MIC_90_ values of β-lactam components for cefepime-taniborbactam, ceftazidime-avibactam, and meropenem-vaborbactam were 2, 2, and 1 mg/L, respectively. For cefepime-taniborbactam, 100% and 99.5% of isolates of *K. pneumoniae* were inhibited at ≤16 mg/L and ≤8 mg/L, respectively, while 98.0% and 95.5% of isolates were susceptible to ceftazidime-avibactam and meropenem-vaborbactam, respectively. For *P. aeruginosa*, MIC_90_ values of β-lactam components of cefepime-taniborbactam, ceftazidime-avibactam, ceftolozane-tazobactam, and meropenem-vaborbactam were 16, >8, >8, and >4 mg/L, respectively. Of 89 carbapenem-susceptible isolates, 100% were susceptible to ceftolozane-tazobactam, ceftazidime-avibactam, and cefepime-taniborbactam at ≤8 mg/L. Of 73 carbapenem-intermediate/resistant *P. aeruginosa* isolates without carbapenemases, 87.7% were susceptible to ceftolozane-tazobactam, 79.5% to ceftazidime-avibactam, and 95.9% and 83.6% to cefepime-taniborbactam at ≤16 mg/L and ≤8 mg/L, respectively. Cefepime-taniborbactam at ≤16 mg/L and ≤8 mg/L, respectively, was active against 73.3% and 46.7% of 15 VIM- and 60.0% and 35.0% of 20 KPC-producing *P. aeruginosa* isolates. Of all 108 carbapenem-intermediate/resistant *P. aeruginosa* isolates, cefepime-taniborbactam was active against 86.1% and 69.4% at ≤16 mg/L and ≤8 mg/L, respectively, compared to 59.3% for ceftolozane-tazobactam and 63.0% for ceftazidime-avibactam. Cefepime-taniborbactam had *in vitro* activity comparable to ceftazidime-avibactam and greater than meropenem-vaborbactam against carbapenem-resistant *K. pneumoniae* and carbapenem-intermediate/resistant MDR *P. aeruginosa*.

## INTRODUCTION

In a recent review by the World Health Organization (WHO) of antimicrobials currently under development, 43 antibiotics and combinations with a new therapeutic entity and 27 non-traditional antibacterial agents were identified (1). Of these, 27 are active against WHO-priority pathogens such as carbapenem-resistant Gram-negative bacilli, 13 against *Mycobacterium tuberculosis,* and five against *Clostridioides difficile*. Twelve of these agents are β-lactam and β-lactamase inhibitor combinations, but it was noted that many are not active against metallo-β-lactamase (MBL) producers. The report also noted that, despite 11 new antibiotics being approved for use since 2017, these agents are from existing classes where resistance mechanisms are well established and have limited clinical benefit over existing treatment. Seven agents fulfilled at least one innovation criterion and two were active against multidrug-resistant (MDR) Gram-negative bacteria. Only two of the seven innovative antibiotics, taniborbactam (formerly VNRX-5133) in combination with cefepime and ledaborbactam (formerly VNRX-7145) in combination with ceftibuten, target at least one of the WHO critical priority antimicrobial-resistant Gram-negative bacterial groups.

β-Lactamase inhibitors have been an important component of antibacterials since the introduction of clavulanate into clinical use in 1984. Three classes of β-lactamase inhibitors are now in clinical use—agents with a β-lactam core (clavulanate, sulbactam, and tazobactam), agents with a diazabicyclooctane (DBO) core (avibactam and relebactam), and agents with a boronic acid core (vaborbactam) (2). Vaborbactam inhibits *Klebsiella pneumoniae* carbapenemase (KPC) and other class A carbapenemases (IMI/NMC and SME), but not class D (OXA) or metallo (class B; IMP, NDM, VIM) types (3). Taniborbactam has been reported to have a broader spectrum of direct inhibition than any other β-lactamase inhibitor in use or in advanced clinical development, with cefepime-taniborbactam having similar coverage of carbapenem-resistant Enterobacterales as β-lactam/DBO combinations, including aztreonam-avibactam, and cefiderocol (4, 5). Taniborbactam is under clinical development in combination with cefepime at its highest approved dose (6 g/day in three divided doses) (6, 7). In a phase 3, double-blind, randomized trial, 661 hospitalized adults with complicated urinary tract infections, including acute pyelonephritis, were randomized in a 2:1 ratio to receive intravenous cefepime-taniborbactam (2.5 g) or meropenem (1 g) every 8 hours for 7 days (or up to 14 days for patients with concurrent bacteriemia) (6). Cefepime-taniborbactam was superior to meropenem regarding the primary composite outcome of both microbiologic and clinical success on trial days 19 to 23, with a treatment difference of 12.6% (95% CI, 3.1%–22.2%; *P* = 0.009). Higher composite success and clinical success rates were sustained at late follow-up (trial days 28 to 35).

This study was undertaken to evaluate the activity of cefepime-taniborbactam against collections of carbapenem-resistant *K. pneumoniae* and MDR *Pseudomonas aeruginosa* with characterized carbapenem resistance mechanisms, including those found in many regions of the United States (8–11).

## RESULTS

### K. pneumoniae

Of the 200 isolates tested, 192 contained a class A *K. pneumoniae* carbapenemase (KPC) and seven contained a class D carbapenem-hydrolyzing oxacillinase (OXA) variant of OXA-48, with one of these seven also containing a class B New Delhi metallo-β-lactamase (NDM). One isolate with CTX-M-15, OXA-1, TEM-1, and SHV-28 β-lactamases had no known carbapenemases. The minimum inhibitory concentration inhibiting 50% or 90% of isolates (MIC_50/90_) and the percentage susceptible to the agents tested are shown in Table 1. As expected, based on most isolates containing carbapenemases, susceptibility to the β-lactam agents tested—ceftazidime, cefepime, and meropenem—was low (<3%). A majority of isolates were susceptible to amikacin (60.0%), and tigecycline (88.5%), with 77.0% intermediate to colistin.

**TABLE 1:** MIC_50/90_ values and percent susceptibility of *K. pneumoniae* isolates (*n* = 200)^*a*^

Agent (susceptible breakpoint, mg/L)	MIC range (mg/L)	MIC_50_ (mg/L)	MIC_90_ (mg/L)	Percent susceptible
Amikacin (≤16)	≤0.5 to >32	16	32	60.0
Colistin (≤2)^*b*^	0.25 to >4	0.5	>4	77.0^*b*^
Ceftazidime (≤4)	0.5 to >16	>16	>16	1.0
Ceftazidime-avibactam (≤8)	≤0.06 to >8	1	2	98.0
Cefepime (≤2)	2 to >16	>32	>32	1.5
Cefepime-taniborbactam (≤8/≤16)^*c*^	≤0.06 to 16	0.25	2	99.5/100^*c*^
Meropenem (≤1)	0.5 to >4	>4	>4	2.5
Meropenem-vaborbactam (≤4)	≤0.06 to 16	≤0.03	1	95.5
Tigecycline (≤2)	0.5 to >4	1	4	88.5

^*a*^ MIC values of β-lactam/β-lactamase inhibitor combinations are shown as MICs of the β-lactam component.

^*b*^ CLSI interpretation is intermediate with no susceptible category (12).

^*c*^ For comparative purposes only, the percent susceptible for cefepime-taniborbactam corresponds to the percentage of isolates inhibited at ≤8 and ≤16 mg/L.

**Fig 1:**
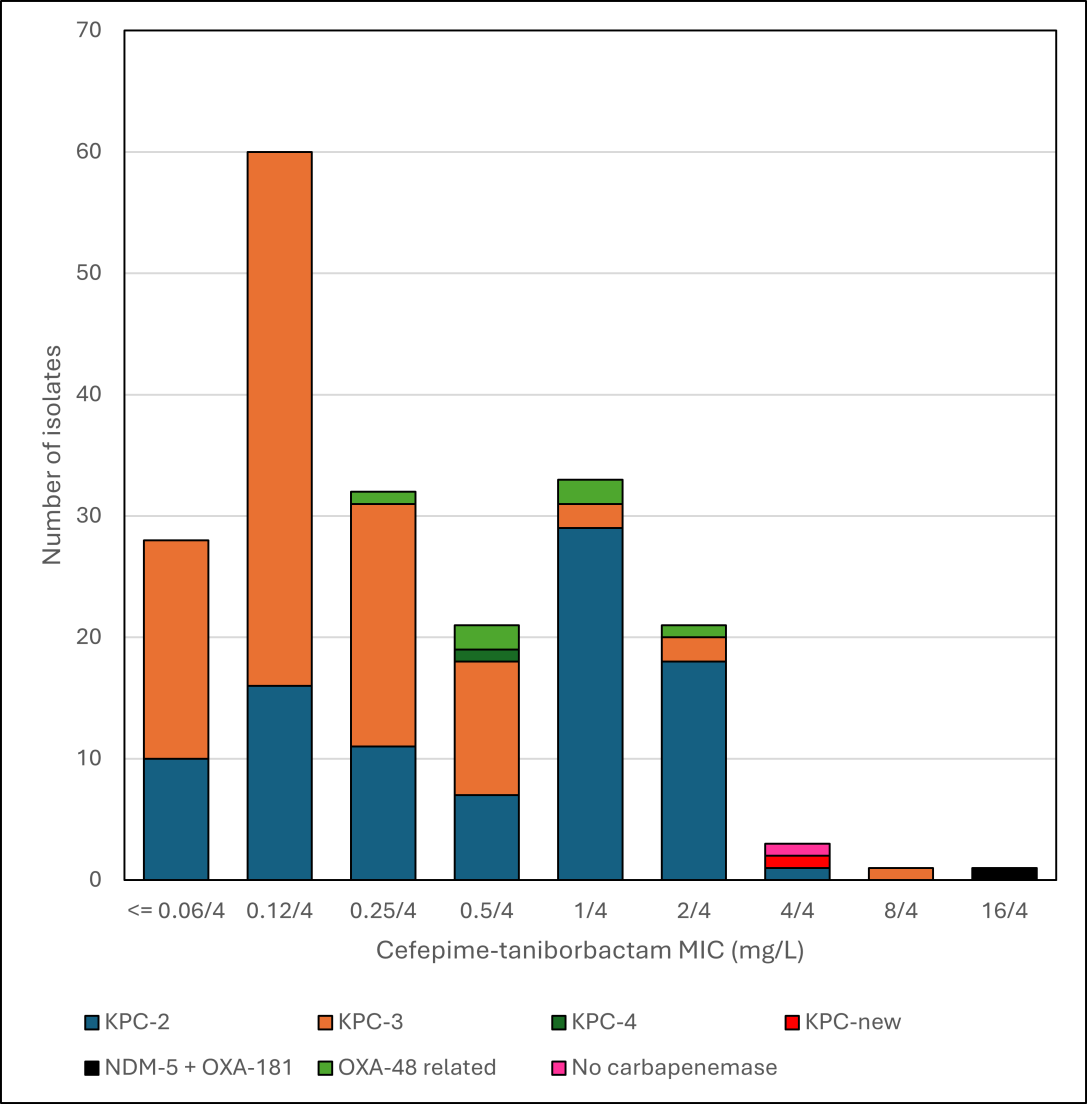
Histogram of cefepime-taniborbactam MICs of *K. pneumoniae* by carbapenemase type.

Susceptibility to the β-lactam/β-lactamase inhibitor combinations tested was high: ceftazidime-avibactam (MIC_50/90_ 1/2 mg/L; 98.0% susceptible), cefepime-taniborbactam (MIC_50/90_ 0.25/2 mg/L; 100% and 99.5% inhibited at ≤16 mg/L and ≤8 mg/L, respectively), and meropenem-vaborbactam (MIC_50/90_ ≤0.03/1 mg/L; 95.5% susceptible) (Table 1). MIC distributions of cefepime and cefepime-taniborbactam are shown in Table 2 and Fig. 1. The five isolates with the highest cefepime-taniborbactam MIC values (ranging from 4 to 16 mg/L) were susceptible at a provisional breakpoint of ≤16 mg/L, which is supported by preclinical and clinical data (13–17). MICs for the β-lactam and β-lactam/β-lactamase inhibitor combinations tested against these five isolates, as well as isolates intermediate/resistant to ceftazidime-avibactam and meropenem-vaborbactam and all OXA-producing isolates, are shown in Table 3 with their β-lactam resistance mechanisms. Twelve of these 13 isolates had mutations in *ompK35* (*n* = 12), *ompK36* (*n* = 7), and/or PBP3 genes (*n* = 1). The *ompK35* mutations included insertion sequences (IS), deletions, and premature stop codons. The *ompK36* mutations included two di-amino acid insertions. The *ftsI* mutation encoded a variant PBP3 (F383Y). Six of the seven OXA-48-related producing isolates were intermediate/resistant to meropenem-vaborbactam, with three KPC-producing isolates also resistant to this combination. Four isolates were intermediate/resistant to ceftazidime-avibactam (one OXA-48-related and three KPC-producing). All 13 isolates were inhibited by cefepime-taniborbactam at ≤16 mg/L, with 11 (84.6%) inhibited at ≤4 mg/L. The lone isolate in this set with a cefepime-taniborbactam MIC of 16 mg/L harbored both an OXA-48 variant and NDM-5.

**TABLE 2:** Distribution of MICs of cefepime alone and in combination with taniborbactam for *K. pneumoniae* and *P. aeruginosa* isolates tested^*a*^

	Agent	Number of isolates with MIC value for agent shown (MIC values in mg/L)												
		≤0.06	0.12	0.25	0.5	1	2	4	8	16	32	64	128	>128
*K. pneumoniae*, all (*n* = 200)	Cefepime	–^*b*^	–	–	–	–	3	3	21	32	141^*c*^	–	–	–
	Cefepime-taniborbactam	28	60	32	21	33	21	3	1	1	–	–	–	–
*P. aeruginosa*, all (*n* = 197)	Cefepime	–	–	–	–	9	64	34	25	10	55^*c*^	–	–	–
	Cefepime-taniborbactam	–	–	–	1	38	55	36	34	18	5	2	2	6
*P. aeruginosa*, carbapenem resistant, all (*n* = 108)	Cefepime	–	–	–	–	–	13	10	21	9	55^*c*^	–	–	–
	Cefepime-taniborbactam	–	–	–	–	8	14	22	31	18	5	2	2	6
*P. aeruginosa*, carbapenem resistant, KPC (*n* = 20)	Cefepime	–	–	–	–	–	–	–	–	–	20^*c*^	–	–	–
	Cefepime-taniborbactam	–	–	–	–	–	–	6	1	5	3	2	1	2
*P. aeruginosa*, carbapenem resistant, VIM (*n* = 15)	Cefepime	–	–	–	–	–	–	–	–	2	13^*c*^	–	–	–
	Cefepime-taniborbactam	–	–	–	–	–	1	2	4	4	–	–	1	3
*P. aeruginosa*, carbapenem resistant, other (*n* = 73)	Cefepime	–	–	–	–	–	13	10	21	7	22^*c*^	–	–	–
	Cefepime-taniborbactam	–	–	–	–	8	13	14	26	9	2	–	–	1
*P. aeruginosa*, carbapenem susceptible (*n* = 89)	Cefepime	–	–	–	–	9	51	24	4	1	–	–	–	–
	Cefepime-taniborbactam	–	–	–	1	30	41	14	3	–	–	–	–	–

^*a*^ MIC values of β-lactam/β-lactamase inhibitor combinations are shown as MICs of the β-lactam component.

^*b*^ No isolates with MIC indicated or concentration not tested.

^*c*^ MIC value greater than the previous MIC value.

**TABLE 3:** β-Lactam resistance mechanisms and MICs of β-lactams and β-lactam/β-lactamase inhibitor agents of *K. pneumoniae* isolates with the highest cefepime-taniborbactam MIC values (≥4 mg/L), intermediate/resistant to one or more of the β-lactam/β-lactamase inhibitor agents and/or producing OXA-48-derived β-lactamases^*a*^

	Resistance mechanisms				MIC (mg/L) by agent					
Isolate no.	β-Lactamases	Porin OmpK35	Porin OmpK36	PBP3^*b*^	Ceftazidime	Ceftazidime- avibactam	Cefepime	Cefepime- taniborbactam	Meropenem	Meropenem- vaborbactam
CRK0103 (ALRG2756)	OXA-181, NDM-5, SHV-11, TEM-1, CTX-M-15	Lesion^*c*^	TD insertion^*i*^	WT^*k*^	>16	>8	>16	16	>4	>4
CRK0102 (ALRG2757)	OXA-181, SHV-11, CTX-M-15	Lesion^*c*^	TD insertion^*i*^	WT	>16	0.5	>16	1	>4	>4
CRK0297 (ALRG2881)	OXA-48, SHV-11, TEM-1	Lesion^*d*^	Intact	WT	0.5	0.25	2	0.5	>4	>4
CRK0298 (ALRG2882)	OXA-48, SHV-11, TEM-1,	Lesion^*d*^	Intact	WT	0.5	0.25	2	0.25	>4	>4
CRK0035 (ALRG2946)	OXA-48, OXA-1, SHV-1, CTX-M-15	Intact	Intact	WT	>16	1	>16	0.5	2	2
CRK0299 (ALRG3126)	OXA-232, SHV-1, TEM-1	Lesion^*e*^	TD insertion^*i*^	WT	>16	1	>16	2	>4	>4
CRK0300 (ALRG3143)	OXA-232, SHV-1, TEM-1	Lesion^*e*^	TD insertion^*i*^	WT	>16	1	>16	1	>4	>4
CRK0188 (ALRG1995)	KPC-2, SHV-12, TEM-1	Lesion^*f*^	GD insertion^*i*^	WT	>16	4	>16	4	>4	>4
CRK0089 (ALRG2019)	KPC-3, SHV-12	Lesion^*f*^	Intact	F383Y	>16	>8	>16	8	>4	0.5
CRK0154 (ALRG2490)	KPC-2, SHV-11	Lesion^*f*^	GD insertion^*i*^	WT	>16	2	>16	2	>4	>4
CRK0114 (ALRG2834)	KPC-4, SHV-1, OXA-1, TEM-1	Lesion^*g*^	Intact	WT	>16	>8	8	0.5	2	≤0.03
CRK0113 (ALRG2835)	KPC-new (KPC-4 W105G), SHV-1	Lesion^*g*^	Lesion^*j*^	WT	>16	>8	>16	4	>4	>4
CRK0080 (ALRG2512)	CTX-M-15, OXA-1, TEM-1, SHV-28	Lesion^*h*^	Intact	WT	>16	1	>16	4	1	0.12

^*a*^ MIC values of β-lactam/β-lactamase inhibitor combinations are shown as MICs of the β-lactam component.

^*b*^ PBP3, penicillin-binding protein 3.

^*c*^ IS insertion at codon 274.

^*d*^ Frameshift by 11-bp deletion starting at codon 254.

^*e*^ Premature stop at codon 107.

^*f*^ 1-bp insertion at codon 40.

^*g*^ 2-bp insertion at codon 20.

^*h*^ 1-bp insertion at codon 179.

^*i*^ Key di-amino acid insertion (TD or GD) in the extracellular loop 3 region that restricts entry of many β-lactams into the bacterial cell (18).

^*j*^ IS insertion at codon 82.

^*k*^ Wild-type.

### P. aeruginosa

**Fig 2:**
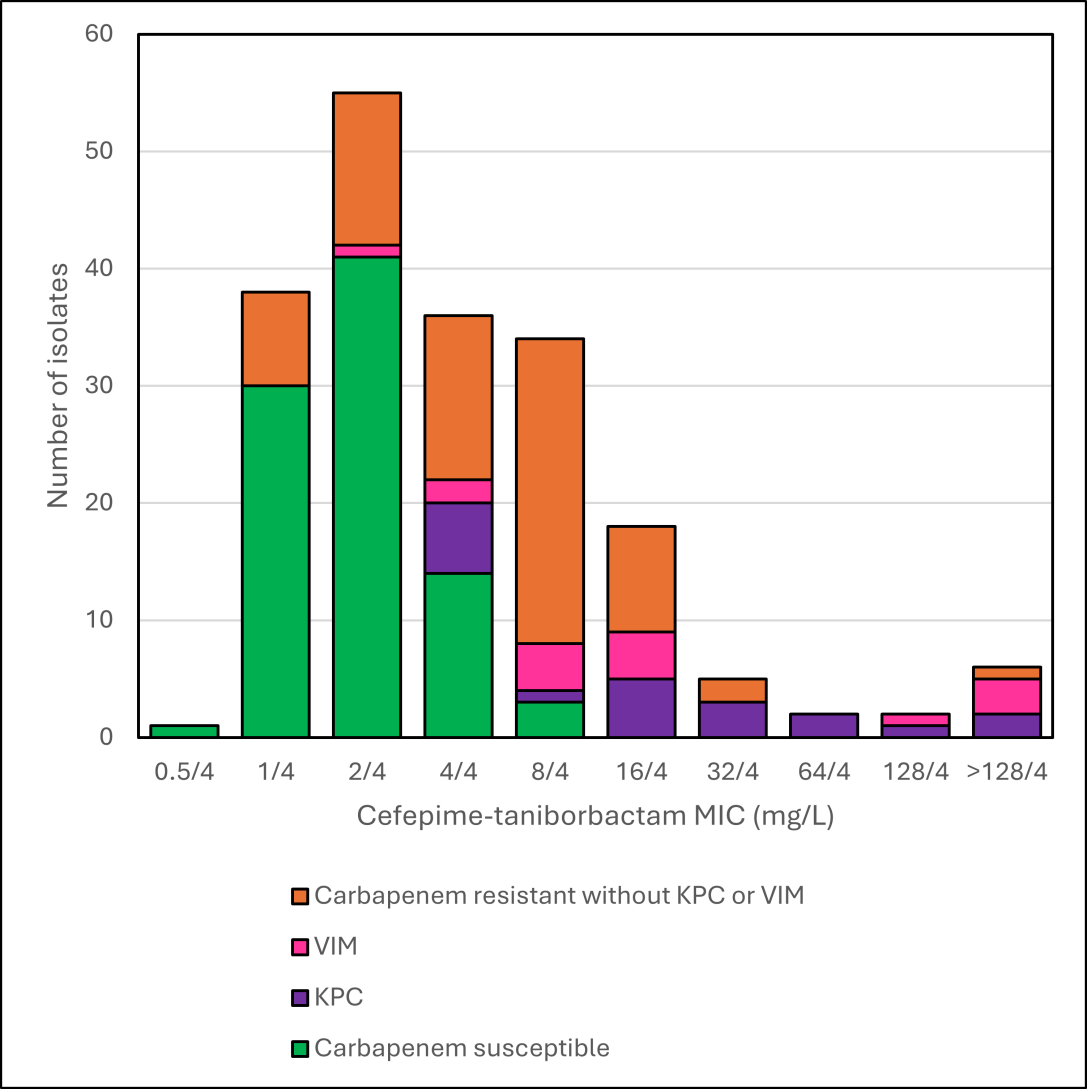
Histogram of cefepime-taniborbactam MICs of *P. aeruginosa* by carbapenem resistance mechanism.

Of the 197 tested MDR isolates of *P. aeruginosa*, 89 were carbapenem-susceptible and 108 were intermediate/resistant, with carbapenem resistance associated with porin changes or efflux pumps (*n* = 73), and with the presence of acquired carbapenemases, KPC (*n* = 20) and VIM (*n* = 15). MIC distributions of cefepime-taniborbactam by carbapenem resistance mechanism are shown in Table 2 and Fig. 2. MIC_50/90_ values and percentage of isolates susceptible to agents tested are shown in Table 4 for all isolates and by carbapenem resistance mechanism. Overall, amikacin (89.3% susceptible) and cefepime-taniborbactam (92.4% and 82.7% inhibited at ≤16 mg/L and ≤8 mg/L, respectively) were the most active agents, followed by ceftazidime-avibactam (79.7%), tobramycin (78.6%), and ceftolozane-tazobactam (77.7%). Greater than 95% of isolates in the carbapenem-susceptible group (*n* = 89) were susceptible to the agents tested, except for aztreonam (88.8% susceptible). In the carbapenem-intermediate/resistant group without KPC or VIM (*n* = 73), amikacin (97.3% susceptible), ceftolozane-tazobactam (87.7%), cefepime-taniborbactam (95.9% and 83.6% inhibited at ≤16 mg/L and ≤8 mg/L, respectively), and tobramycin (82.2%) were the most active agents, and these four agents were also the most active against all carbapenem-intermediate/resistant isolates (*n* = 108). In the KPC group (*n* = 20), only amikacin (55.0% susceptible), ceftazidime-avibactam (50.0%), and cefepime-taniborbactam (60.0% and 35.0% inhibited at ≤16 mg/L and ≤8 mg/L, respectively) had any activity among the tested agents. In the VIM group (*n* = 15), only cefepime-taniborbactam (73.3% and 46.7% inhibited at ≤16 mg/L and ≤8 mg/L, respectively), aztreonam (33.3%), and amikacin (6.7%) had any activity. MICs of the β-lactams and β-lactam/β-lactamase inhibitor combinations tested for isolates with KPC (*n* = 11) or VIM (*n* = 7) and susceptible to one or more of the β-lactam/β-lactamase inhibitor agents are shown in Table 5. In this isolate set (*n* = 18), cefepime-taniborbactam (83.3% and 77.8% inhibited at ≤16 mg/L and ≤8 mg/L, respectively) was the most active agent, followed by ceftazidime-avibactam (55.6% susceptible) and aztreonam (16.7% susceptible, *n* = 3 isolates, all producing VIM). All isolates with VIM were resistant to ceftazidime-avibactam, while 10 of the 11 isolates with KPC were susceptible. Cefepime-taniborbactam was active against 8 of the 11 isolates with KPC and all 7 with VIM.

**TABLE 4:** MIC_50/90_ values and percent susceptibility of *P. aeruginosa* isolates (*n* = 197)^*a*^

Agent (susceptible breakpoint, mg/L)	All (*n* = 197)		Carbapenem susceptible (*n* = 89)		Carbapenem intermediate/ resistant, KPC (*n* = 20)		Carbapenem intermediate/ resistant, VIM (*n* = 15)		Carbapenem intermediate/ resistant, other (*n* = 73)		All carbapenem intermediate/ resistant (*n* = 108)	
	MIC_50/90_	%S	MIC_50/90_	%S	MIC_50/90_	%S	MIC_50/90_	%S	MIC_50/90_	%S	MIC_50/90_	%S
Amikacin (≤16)	4/64	89.3	4	100.0	16/>32	55.0	>32/>32	6.7	4/16	97.3	4/>32	76.9
Aztreonam (≤8)	8/>16	53.8	4/16	88.8	>16/>16	0.0	16/>16	33.3	>16/>16	38.4	>16/>16	25.0
Ceftolozane-tazobactam (≤4)	0.5/>8	77.7	0.5/1	100.0	>8/>8	0.0	>8/>8	0.0	1/8	87.7	4/>8	59.3
Ceftazidime (≤8)	4/>16	60.9	2/8	97.8	>16/>16	0.0	>16/>16	0.0	16/>16	45.2	>16/>16	30.6
Ceftazidime-avibactam (≤8)	2/>8	79.7	2/4	100.0	8/>8	50.0	>8/>8	0.0	4/>8	79.5	8/>8	63.0
Cefepime (≤8)	4/>32	67.0	2/4	98.9	>16/>16	0.0	>16/>16	0.0	8/>16	60.3	>16/>16	40.7
Cefepime-taniborbactam (≤8)^*b*^	4/16	82.7	2/4	100.0	16/128	35.0	4/>128	46.7	8/16	83.6	8/32	69.4
Cefepime-taniborbactam (≤16)^*b*^	4/16	92.4	2/4	100.0	16/128	60.0	4/>128	73.3	8/16	95.9	8/32	86.1
Imipenem (≤2)	4/>4	45.2	1/2	100.0	>4/>4	0.0	>4/>4	0.0	>4/>4	0.0	>4/>4	0.0
Meropenem (≤2)	1/>4	55.8	0.25/0.5	100.0	>4/>4	0.0	>4/>4	0.0	>4/>4	28.8	>4/>4	19.4
Meropenem-vaborbactam^*c*^	1/>4	–^*d*^	0.12/0.5	–	>4/>4	–	>4/>4	–	>4/>4	–	>4/>4	–
Piperacillin-tazobactam (≤16)	8/>64	56.8	4	97.8	>64/>64	0.0	>64/>64	0.0	32/>64	34.2	>64/>64	23.1
Tobramycin (≤4)	0.5/>8	78.6	0.5/1	100.0	>8/>8	0.0	>8/>8	0.0	0.5/>8	82.2	1/>8	61.1

^*a*^ MIC values of β-lactam/β-lactamase inhibitor combinations are shown as MICs of the β-lactam component.

^*b*^ For comparative purposes only, the percent susceptible for cefepime-taniborbactam corresponds to the percentage of isolates inhibited at ≤8 and ≤16 mg/L.

^*c*^ No CLSI breakpoint is available.

^*d*^ Not applicable.

**TABLE 5:** MICs of β-lactam and β-lactam/β-lactamase inhibitor combinations of 18 *P*. *aeruginosa* isolates with KPC or VIM and susceptible to one or more of the β-lactam/β-lactamase inhibitor agents^*a*^^,^^*b*^

Agent (susceptible breakpoint, mg/L)	MICs (mg/L) of 11 KPC-producing isolates											MICs (mg/L) of 7 VIM-producing isolates							Percent susceptible
Aztreonam (≤8)	>16	>16	>16	>16	>16	>16	>16	>16	>16	>16	>16	>16	16	4	4	16	16	8	16.7
Ceftolozane-tazobactam (≤4)	>8	>8	8	8	>8	>8	8	>8	>8	>8	8	>8	>8	>8	>8	>8	>8	>8	0
Ceftazidime (≤8)	>16	>16	>16	>16	>16	>16	>16	>16	>16	>16	>16	>16	>16	>16	>16	>16	>16	>16	0
Ceftazidime-avibactam (≤8)	8	8	1	8	4	>8	4	8	1	8	2	>8	>8	>8	>8	>8	>8	>8	55.6
Cefepime (≤8)	>16	>16	>16	>16	>16	>16	>16	>16	>16	>16	>16	16	>16	>16	>16	16	>16	>16	0
Cefepime-taniborbactam (≤8/≤16)^*c*^	4	32	4	16	4	4	128	>128	4	4	8	4	8	2	8	8	4	8	77.8/83.3
Meropenem (≤2)	>4	>4	>4	>4	>4	>4	>4	>4	>4	>4	>4	4	>4	>4	>4	>4	>4	>4	0
Meropenem-vaborbactam^*d*^	4	>4	>4	>4	>4	>4	>4	>4	>4	>4	>4	>4	>4	>4	>4	>4	>4	4	–^*e*^

^*a*^ MIC values of β-lactam/β-lactamase inhibitor combinations are shown as MICs of the β-lactam component; shaded results indicate MICs in the susceptible range.

^*b*^ All other KPC- (*n* = 9) and VIM-producing isolates (*n* = 8) were intermediate/resistant to aztreonam and the β-lactam/β-lactamase inhibitor agents tested.

^*c*^ For comparative purposes only, the percent susceptible for cefepime-taniborbactam corresponds to the percentage of isolates inhibited at ≤8 and ≤16 mg/L.

^*d*^ No CLSI breakpoint is available.

^*e*^ Not applicable.

## DISCUSSION

In a recent review of eight β-lactam/β-lactamase inhibitors in development, the authors concluded that these agents provide various levels of *in vitro* coverage of carbapenem-resistant Enterobacterales, with only cefepime-zidebactam, aztreonam-avibactam, meropenem-nacubactam, and cefepime-taniborbactam showing *in vitro* activity against isolates producing MBLs (19). Cefepime-zidebactam and cefepime-taniborbactam were also noted to have *in vitro* activity against carbapenem-resistant *P. aeruginosa*, and cefepime-zidebactam and sulbactam-durlobactam against carbapenem-resistant *Acinetobacter baumannii* (19). Among these β-lactamase inhibitors, only taniborbactam has been shown *in vitro* to inhibit class B metallo-β-lactamases (NDM, VIM, and SPM, but not IMP) (4, 5).

Recent publications have documented the *in vitro* activity of cefepime-taniborbactam. A study by Mushtaq et al. showed that cefepime-taniborbactam was active against Enterobacterales with KPC, other class A, OXA-48-like, VIM, and NDM carbapenemases, with MICs similar to those of ceftazidime-avibactam for Enterobacterales with KPC or OXA-48-like carbapenemases and a wider spectrum (3). Against the subset Enterobacterales with NDM (*n* = 124), cefepime-taniborbactam inhibited 89 (71.8%) isolates at ≤8 mg/L and 98 (79.0%) at ≤16 mg/L compared to <1% susceptible to ceftazidime-avibactam and meropenem-vaborbactam. The cefepime-taniborbactam MIC_90_ value for Enterobacterales with KPC in that study (0.5 mg/L) was similar to that in our study (2 mg/L). In a study by Wang et al., taniborbactam was reported to improve the activity of cefepime to the same level that avibactam improved the activity of ceftazidime against 66 KPC-2 producers and 30 non-carbapenemase-producing carbapenem-intermediate/resistant Enterobacteriaceae (20). Vazquez-Ucha et al. reported cefepime-taniborbactam MIC_90_ of 2 mg/L against a collection of 400 carbapenemase-producing Enterobacterales, noting that activity was excellent against OXA-48- and KPC-producing Enterobacterales but lower against MBL-producing isolates (21). Kloezen et al*.* found that cefepime-taniborbactam demonstrated potent activity against ESBL-producing Enterobacterales, restoring the susceptibility of all isolates tested (22). Karlowsky et al*.* tested the *in vitro* activity of cefepime-taniborbactam and comparators against a 2018–2020 collection of 13,731 Enterobacterales and 4,619 *P*. *aeruginosa* isolated from patients in 56 countries (23). This study showed that cefepime-taniborbactam exhibited potent *in vitro* activity against Enterobacterales and *P. aeruginosa* and inhibited most carbapenem-resistant isolates, including isolates carrying serine carbapenemases (KPC) or metallo-β-lactamases (NDM or VIM). Against Enterobacterales, MIC_90_ was 0.25/4 mg/L, with 99.7% susceptible at a breakpoint of ≤16/4 mg/L. Against *P. aeruginosa*, MIC_90_ was 8 mg/L, with 97.4% susceptible at a breakpoint of ≤16/4 mg/L.

Our study provides additional data showing that the activity of cefepime-taniborbactam is comparable to that of ceftazidime-avibactam and meropenem-vaborbactam against carbapenem-resistant, predominantly KPC-producing, *K. pneumoniae* (100% of isolates inhibited at ≤16 mg/L; MIC_50/90_ 0.25/2 mg/L) (Table 1). Cefepime-taniborbactam was active (MICs 0.5 to 16 mg/L) against all study isolates, including 10 isolates intermediate/resistant to one or more of the other β-lactam/β-lactamase inhibitor agents tested (Table 3). These isolates had several resistance mechanisms, including OXA-48-derived β-lactamases, mutations in the major porin genes *ompK35* and *ompK36*, associated with decreased drug permeability (18), or the *ftsI* (PBP3) gene, associated with decreased cefepime binding (24) (Table 3). Mutations in the porin gene *ompK35* included IS insertion at codon 274 (*n* = 2), frameshift by 11 bp deletion starting at codon 254 (*n* = 2), premature stop at codon 107 (*n* = 2), 1 bp insertion at codon 40 (*n* = 3), 2 bp insertion at codon 20 (*n* = 2), and 1 bp insertion at codon 179 (*n* = 1). Mutations in porin gene *ompK36* included TD or GD insertion in the extracellular loop (*n* = 6) and IS insertion at codon 82 (*n* = 1). An F383Y mutation in the *ftsI* (PBP3) gene was present in one isolate.

A limitation of our study is that the isolates contained a limited spectrum of β-lactamases other than KPC. Only seven isolates produced OXA-48-derived β-lactamases, with one of these coproducing a MBL (NDM-5).

Our study also shows that the activity of cefepime-taniborbactam is comparable to that of ceftolozane-tazobactam and ceftazidime-avibactam against carbapenem-resistant MDR *P. aeruginosa* not associated with carbapenemases (95.9%/83.6% of isolates susceptible at ≤16 mg/L/≤8 mg/L) and has activity against some VIM-producing isolates (73.3%/46.7% of isolates inhibited at ≤16 mg/L/≤8 mg/L) (Table 4). Both ceftazidime-avibactam (50% susceptible) and cefepime-taniborbactam (60%/35% inhibited at ≤16 mg/L/≤8 mg/L) had activity against some KPC-producing *P. aeruginosa* isolates. According to ongoing Centers for Disease Control and Prevention (CDC) surveillance, non-carbapenemase-producing *P. aeruginosa* overwhelmingly represents the most prevalent genotype among carbapenem-resistant *P. aeruginosa* in the United States, where 97.8% of 68,172 isolates tested as of June 2023 did not carry a carbapenemase gene (25). In the present study, cefepime-taniborbactam at ≤16 mg/L inhibited 70/73 (95.9%) of carbapenem-intermediate/resistant, non-carbapenemase-producing *P. aeruginosa* isolates, a greater proportion than the percentage of these isolates that were susceptible to ceftolozane-tazobactam (87.7%) and ceftazidime-avibactam (79.5%).

In conclusion, cefepime-taniborbactam was active against most isolates in a set of carbapenem intermediate/resistant *K. pneumoniae* and *P. aeruginosa* isolates with carbapenem resistance mechanisms representative of those currently found in these species in the United States. Cefepime-taniborbactam was very active against KPC-producing *K. pneumoniae*.

## MATERIALS AND METHODS

*K. pneumoniae* study isolates included 200 clinical isolates collected from 2012–2016 in the Great Lakes Region as part of the Antibacterial Resistance Leadership Group (ARLG) Consortium on Resistance against Carbapenems in *Klebsiella* (CRACKLE-I) Study (26). All isolates had previously been characterized as being carbapenem-resistant, with 199 containing a known carbapenemase determined from whole-genome sequencing (WGS) (NCBI BioProject PRJNA339843 and PRJNA433394). One isolate contained CTX-M-15, OXA-1, TEM-1, and SHV-28 β-lactamases.

*P. aeruginosa* study isolates included 197 MDR clinical *P. aeruginosa* isolates collected as part of the Platforms for Rapid Identification of MDR-Gram-negative bacteria and Evaluation of Resistance Studies IV (PRIMERS-IV) study (11). These isolates came primarily from northeast Ohio and the mid-Atlantic states. Approximately half had been determined to be carbapenem-resistant when initially characterized. Isolates had been tested using two molecular diagnostic platforms, the Acuitas Resistome test (OpGen Inc., Gaithersburg, MD) and VERIGENE Gram-negative blood culture test (VERIGENE BC-GN, Luminex Corporation, Austin, TX), to identify genes that potentially confer resistance associated with porins, efflux pumps, and the presence of acquired carbapenemases (KPC and VIM).

MICs of cefepime-taniborbactam and comparators were determined using Sensititre custom frozen panels (ThermoFisher, Cleveland, OH) inoculated using cation-supplemented Mueller-Hinton broth (ThermoFisher). Taniborbactam was supplied by Venatorx Pharmaceuticals Inc. (Malvern, PA). In-house frozen panels were used to obtain cefepime-taniborbactam MIC endpoints of up to 128 mg/L for isolates with MICs of >8 mg/L from the initial panel. β-Lactamase inhibitors were tested at fixed concentrations in combination with varying concentrations of β-lactams, with avibactam, tazobactam, taniborbactam, and tazobactam tested at 4 mg/L and vaborbactam at 8 mg/L. Panels were inoculated according to standard methods and were read visually after incubation as previously described (9, 10). MICs were interpreted using CLSI M100-S30 standard (12), with two exceptions. Meropenem-vaborbactam MICs for *P. aeruginosa* were not interpreted due to a lack of applicable breakpoints in the CLSI M100-S30 standard. Cefepime-taniborbactam MICs against both *Enterobacterales* and *P. aeruginosa* were interpreted using provisional breakpoints of ≤8 mg/L and ≤16 mg/L (susceptible) and >16 mg/L (resistant) (23), based upon *in vivo* efficacy data from neutropenic murine infection models (thigh, complicated urinary tract, lung) (13, 14, 17) and data from safety and pharmacokinetics studies in human volunteers (15, 16). Quality control isolates *E. coli* ATCC 25922, *E. coli* ATCC 35218, *K. pneumoniae* ATCC 700603, *K. pneumoniae* ATCC BAA-1705, and *P. aeruginosa* ATCC 27853 were tested on each day of testing.

MIC values were analyzed and MIC distributions, categorical susceptibility, and MIC_50_ and MIC_90_ values were determined. Categorical susceptibility results were classified into two groups—susceptible and intermediate/resistant, the latter including intermediate, susceptible dose-dependent, and resistant interpretations. MIC values of β-lactam/β-lactamase inhibitor combinations are shown as MICs of the β-lactam component.

The genetic basis of β-lactamase, porin *ompK35* and *ompK36*, and *ftsI* (penicillin-binding protein 3, PBP3) genes in isolates of *K. pneumoniae* intermediate/resistant to one or more of the β-lactam/β-lactamase inhibitor agents tested or producing OXA-48-derived β-lactamases was determined by analysis of results of previously performed WGS (26) with reference genomes as follows. WGS analysis from the genome assembly and FASTQ files (NCBI BioProject PRJNA339843 for CRK0001-CRK0191 and PRJNA433394 for CRK0192-CRL0390) was performed using Geneious Prime version 2023.2 (Biomatters Inc., Boston, MA, USA). β-Lactamases in each genome were annotated using a search set of 90 representative β-lactamases with a cut-off of 40% identity and BLAST search at the Beta-Lactamase DataBase (BLDB) (http://www.bldb.eu/) (27). This search set identifies all ∼2,000 β-lactamases included in ResFinder 4.1 (28). The genes encoding major porins (*ompK36* and *ompK35*) and the *ftsI* gene encoding PBP3 (the major target of cefepime) were analyzed using the gene sequences of a reference genome (Nucleotide accession NZ_KN046818.1).
